# Uptake of Dimethylsulfoniopropionate (DMSP) by Natural Microbial Communities of the Great Barrier Reef (GBR), Australia

**DOI:** 10.3390/microorganisms9091891

**Published:** 2021-09-06

**Authors:** Eva Fernandez, Martin Ostrowski, Nachshon Siboni, Justin R. Seymour, Katherina Petrou

**Affiliations:** 1 School of Life Sciences, University of Technology Sydney, Ultimo, NSW 2007, Australia; Eva.Fernandez@alumni.uts.edu.au; 2 Climate Change Cluster, University of Technology Sydney, Ultimo, NSW 2007, Australia; Martin.Ostrowski@uts.edu.au (M.O.); Nahshon.Siboni@uts.edu.au (N.S.); Justin.Seymour@uts.edu.au (J.R.S.)

**Keywords:** phytoplankton, bacteria, sulfur cycling, dimethyl sulfide, coral reefs

## Abstract

Dimethylsulfoniopropionate (DMSP) is a key organic sulfur compound that is produced by many phytoplankton and macrophytes and is ubiquitous in marine environments. Following its release into the water column, DMSP is primarily metabolised by heterotrophic bacterioplankton, but recent evidence indicates that non-DMSP producing phytoplankton can also assimilate DMSP from the surrounding environment. In this study, we examined the uptake of DMSP by communities of bacteria and phytoplankton within the waters of the Great Barrier Reef (GBR), Australia. We incubated natural GBR seawater with DMSP and quantified the uptake of DMSP by different fractions of the microbial community (>8 µm, 3–8 µm, <3 µm). We also evaluated how microbial community composition and the abundances of DMSP degrading genes are influenced by elevated dissolved DMSP levels. Our results showed uptake and accumulation of DMSP in all size fractions of the microbial community, with the largest fraction (>8 µm) forming the dominant sink, increasing in particulate DMSP by 44–115% upon DMSP enrichment. Longer-term incubations showed however, that DMSP retention was short lived (<24 h) and microbial responses to DMSP enrichment differed depending on the community carbon and sulfur demand. The response of the microbial communities from inside the reef indicated a preference towards cleaving DMSP into the climatically active aerosol dimethyl sulfide (DMS), whereas communities from the outer reef were sulfur and carbon limited, resulting in more DMSP being utilised by the cells. Our results show that DMSP uptake is shared across members of the microbial community, highlighting larger phytoplankton taxa as potentially relevant DMSP reservoirs and provide new information on sulfur cycling as a function of community metabolism in deeper, oligotrophic GBR waters.

## 1. Introduction

Dimethylsulfoniopropionate (DMSP) is an important and ubiquitous organic sulfur compound in the marine environment [1]. Phytoplankton are the dominant producers of DMSP, although intracellular concentrations vary from species to species, ranging from extremely low in cyanobacteria and most diatoms to very high, ~300 pg DMSP cell^−1^, in haptophytes and dinoflagellates [2,3,4,5]. In DMSP-producing phytoplankton, DMSP is central to cell metabolism, satisfying up to 71% of the sulfur and 15% of the cells carbon demand [3,6]. Likewise, for DMSP-associated bacterioplankton, DMSP can account for up to 95% of the sulfur and 15% of the carbon demand [7]. Several physiological roles have been attributed to DMSP, including osmoregulation, cryoprotection and antioxidation [8,9,10], while ecologically, DMSP has been shown to function as a grazing deterrent and have bacteriocidal properties [11,12,13].

Intracellular or particulate DMSP (DMSPp) is transported from the synthesising cell into the external seawater environment via exudation, viral lysis or cell senescence [14,15,16]. Once in the water column, dissolved DMSP is readily taken up by the bacterial community [17,18]. Following bacterial uptake, DMSP can be metabolised via two different pathways [19]. The first of these is the demethylation pathway [20,21], which involves the transformation of DMSP into methanethiol (MeSH) and is utilised for cellular energy and protein production [22,23]. The second pathway, known as the cleavage pathway [19], splits DMSP into the climatically active aerosol dimethyl sulfide (DMS) and acrylate, or other carbon-containing molecules depending on the Ddd derivative [24,25]. It has been hypothesised, in what has been termed the ‘bacterial switch’, that bacteria will preferentially utilise the demethylation pathway when there is a need to produce protein for growth, and once that demand is satisfied, they will switch to using the lyase pathway, catabolising the remaining DMSP to DMS [21,26,27]. This ‘switching’ mechanism has recently been shown to work concurrently rather than mutually exclusively in bacteria and is driven by DMSP availability [27], suggesting that both pathways can act in concert, but the relative reliance on each will differ depending on DMSP concentration and metabolic conditions.

Several genes have been identified as key DMSP degraders in the marine environment. The gene DmdA, is the first cataboliser in the demethylation pathway and is the most abundant DMSP degrading gene in the oceans, estimated to be present in 37–58% of all bacterioplankton [28,29]. In contrast, Ddd genes, responsible for DMSP-lyase, are present in less than 10% of bacterioplankton, with the most abundant lyase gene (DddP) present in only 6% of all marine bacterioplankton [30]. This large discrepancy in the abundance of the dominant genes for DMSP degrading pathways has led to an estimation that 80% of DMSP degrades through demethylation [19]. Moreover, DmdA is not only the most abundant DMSP degrading gene, but is also widespread among marine bacteria including Roseobacter, SAR11 and Gammaproteobacteria, while DddP occurs mostly in Roseobacter and SAR116 clades [30,31]. Importantly, SAR11, abundant throughout the world’s oceans, has been shown to produce methanothiol (MeSH) via demethylation, while also cleaving as much as 59% of DMSP uptake to DMS via DddK lyase, highlighting the substantive role this bacterial group plays in marine sulfur cycling [32].

In addition to bacterioplankton, very low or non-DMSP producing phytoplankton have been shown to play a role in DMSP cycling. Prokaryotic phytoplankton from the groups *Prochlorococcus*, *Synechococcus*, as well as some eukaryotic phytoplankton from the dinoflagellates, cryptophytes and diatoms groups have been shown to take up dissolved DMSP [33,34,35,36]. However, to date, the proportion and magnitude of DMSP taken up by phytoplankton in natural communities remains unclear. Furthermore, while there is some indication that diatoms may use DMSP as an antioxidant [37], the utilisation of DMSP by these non-DMSP synthesisers has not been firmly established and no genes for demethylation have yet been found in these organisms [38]. Identifying and describing the uptake and potential catabolism of DMSP by non-DMSP producing phytoplankton species would help complete our understanding of DMSP cycling and potentially DMS production in our oceans [1,26,39], which could improve cloud albedo models and climate projections that currently only account for few phytoplankton functional groups known to be critical to the production of DMS [40,41].

In this study, we examined the uptake of DMSP by natural microbial communities of the Coral Sea surrounding the Great Barrier Reef (GBR) in Australia. These waters are characterised by high solar radiation, low nutrient concentrations and often dominated by cyanobacteria [42,43,44,45], non-DMSP producing phytoplankton. Coral reefs are considered hotspots of DMS and DMSP production [46,47,48,49]. This is mainly attributed to reef-building corals that harbour symbiotic dinoflagellates from the family Symbiodiniaceae, considered the most prolific of all DMSP producers (up to 3831 fmol DMSP cell^−1^ [47]). Moving away from shallow reefs however, DMSP levels are generally low, due to the dilution with continental slope waters, giving rise to the question of the fate and function of DMSP in deep water tropical ecosystems. To better understand microbial DMSP sinks and sources within the oligrotrophic waters of the GBR, we investigated DMSP uptake among natural microbial communities from two distinct reef locations, with the aim to quantify and compare the uptake of DMSP by the bacterial and phytoplankton communities using a size fractionation approach and to evaluate DMSP-induced changes in microbial composition and DMSP-degradation gene abundance over time.

## 2. Materials and Methods

### 2.1. Study Site and Experimental Design

**Figure 1:**
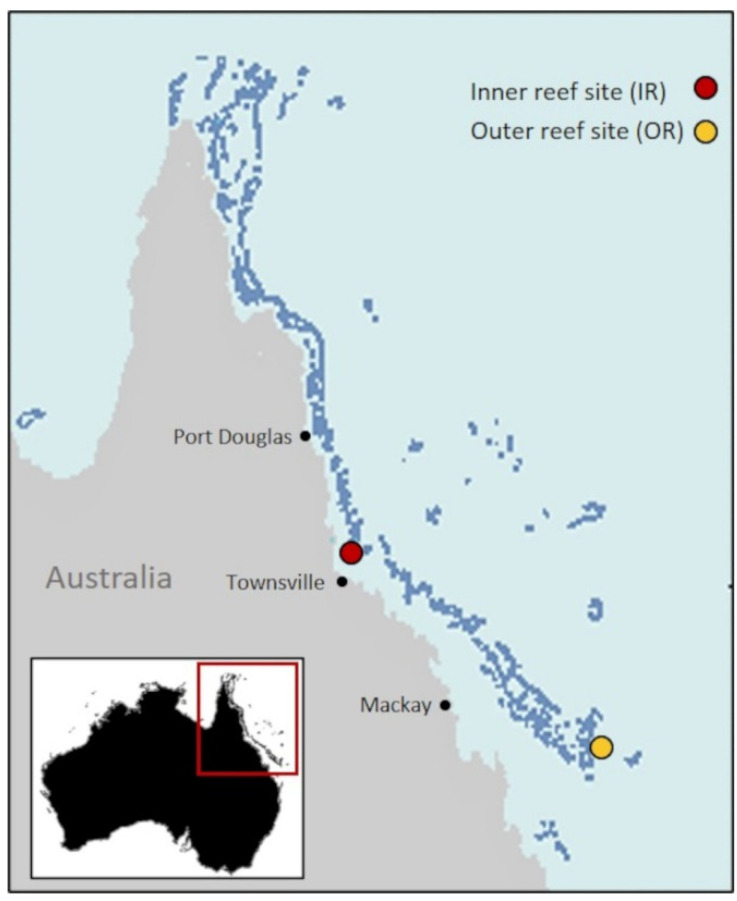
Location of sampling sites of initial water for experiments 1 and 2. Map of Australia showing location of the Great Barrier Reef, with magnified inset image showing location of the inner reef (red dot) and outer reef (yellow dot) sampling sites. Map template provided by Geoscience Australia.

This study was conducted during an oceanographic voyage on the RV Investigator in October 2016. Two sites within the Great Barrier Reef were chosen: a southern open ocean (depth ~400 m) site (outer reef site; OR) (−20°8′ S, 153°1′ E) and a shallower (~45 m) northern inner-reef site (−18°6′ S, 146°9′ E), located between the coast and the reefs (inner reef site; IR). The inner reef site was located ~13 km from the closest reefs of John Brewer Reef, Rib Reef and Fore and Aft Reefs; the deeper outer reef was located ~25 km from Bills Reef (Figure 1). At both sites, surface seawater samples (5 m) were collected with Niskin bottles from CTD casts and filtered through a 210 µm mesh to remove large grazers before being sub-sampled into experimental units. Samples for nutrients, DMS and microbial communities (16S and 18S) were taken from initial samples prior to incubations. Seawater temperature and salinity were taken from the CTD data.

At each site, two separate incubation experiments were conducted: (1) a short-term study (7 h) to quantify DMSP uptake by different size fractions of the microbial community, and (2) a longer (120 h) experiment to investigate the influence of DMSP on community structure and DMSP degradation gene regulation.

### 2.2. Experiment 1: Quantification of DMSP Uptake by Size Fractions of the Microbial Community

To study the uptake of DMSP by the microbial community of the Great Barrier Reef, 28 L of seawater was collected and transferred to 4 L-polycarbonate bottles with no headspace. Bottles were separated into three treatments, in triplicate: controls (unamended seawater), DMSP addition (+10 nM DMSP) and fixed (dead). The fixed sample was incubated with glutaraldehyde (final concentration 1%) for 1 h, before DMSP (10 nM final concentration) was added, to control for any passive uptake of DMSP by the cells. Following DMSP addition, the bottles were closed with screw caps and inverted gently to dissolve the added compound. All flasks were then incubated for up to 7 h in an on-deck flow-through incubator. At four time points (T0, T2, T4 and T7 h) each bottle was subsampled in triplicate for quantification of DMSP total and dissolved (DMSPt, DMSPd) concentrations, either as a whole water sample or a gravity filtered (GF/F, nominal pore size 0.7 µm) sample (3 mL), respectively. Sub-samples were acidified with H_2_SO_4_ (0.5%, pH ~1.1) and kept at −80 °C until processing [50]. After 7 h, two litres from each replicate was size-fractionated via serial filtration onto 8, 3 and 0.22 μm polycarbonate filters for analysis of particulate DMSP (DMSPp) and the filters were snap frozen and kept at −80 °C until analysis. Filters were defrosted and hydrolysed with NaOH prior to GC-FPD analysis, described below. During filtration, aliquots (800 µL) were taken from the whole water fraction, fixed with glutaraldehyde (final concentration 1%) and snap frozen for determination of microbial abundance via flow cytometry (see below).

### 2.3. Experiment 2: Effect of DMSP on Community Structure and Gene Regulation

To study microbial community changes caused by DMSP enrichment, 30 L of water were divided across 15 × 2 L polycarbonate bottles (no headspace). Bottles were separated into two treatments: six controls (unamended) and nine amended with DMSP (10 nM final concentration). Bottles were then incubated in an on-deck flow-through incubator until sampling at three time points (T24, T72 and T120 h). At each time point, subsamples (3 mL) for DMSP total and dissolved (gravity filtration, GF/F) were taken and acidified as described above.

### 2.4. Quantification of Biogenic Sulfur Compounds

Biogenic sulfur compounds (DMSPt, DMSPd) were quantified as total DMS after conversion with 100 mg of NaOH and measured using a gas chromatograph (GC-2010 Plus, Shimadzu, Kyoto, Japan) coupled with a flame photometric detector (FPD). Samples were purged with He (60 mL/min for 4 min) while cryo-trapped in liquid N_2_ and subsequently eluted onto a capillary column (DB-1, Agilent; injector: 120 °C, column: 110 °C, FPD: 130 °C, column flow: 2.1 mL min^−1^). For experiment 1, particulate DMSP (DMSPp) was measured directly on the filtered size fractions, where filters were placed into vials with NaOH and quantified as total DMS as described above. Particulate DMSP for experiment 2 was calculated by subtracting DMSPd from DMSPt. Integrated peak areas for each sample were quantified against a calibration curve obtained from solutions prepared with known amounts of DMSP hydrolysed with NaOH and analysed using the same method as the samples. For determination of DMSP lyase activity (DLA) rates of the starting communities, subsamples (600 mL) were filtered first through a GF/C filter (nominal pore size 1.2 μm) for the phytoplankton fraction rates (DLAp), and then 300 mL of the phytoplankton-free sample filtrate was filtered onto a 0.22 μm polycarbonate filter for bacterial DLA (DLAb). Both filters were placed into cryotubes, flash frozen with in liquid nitrogen and stored in −80 °C until analysis. DMSP lyase activity was determined via direct injection of 500 µL of headspace (column flow: 3.66 mL min^−1^, FPD: 160 °C) as previously described [51].

### 2.5. Quantification of Microbial Composition

Quantification of microbial populations via flow cytometry was performed using a Beckman Coulter Inc flow cytometer (Indianapolis, IN, USA). Populations of *Prochlorococcus*, *Synechococcus* and picoeukaryotes were discriminated using side scatter (SSC) and red and orange fluorescence [52]. Samples for quantification of heterotrophic bacteria were stained with SYBR Green I nucleic acid stain (1:10,000 final dilution; Invitrogen, Waltham, MA, USA) and populations were discriminated according to green fluorescence and side scatter properties [53,54]. Data were analysed using CytExpert software (Beckman Coulter, Brea, CA, USA).

### 2.6. Nutrients

Nutrients, measured in the initial sample (i.e., at time of collection) and at T24, T72 and T120 h from the incubations were carried out on board as previously described [55]. Data were subsequently obtained from the Australian Ocean Data Network (AODN).

### 2.7. Microbial Community Composition and DMSP-Related Genes

In both incubation experiments, the remaining sample (~1.9 L) at the final time-point was filtered through a 0.22 µm polycarbonate filter, which was subsequently snapped frozen and kept at −80 °C. DNA was extracted using the Powerwater DNA isolation kit (QIAGEN, Hilden, Germany) following manufacturer’s instructions, and the DNA concentration was quantified using a Nanodrop spectrophotometer (Nanodrop 2000, Thermo Fisher Scientific, Waltham, MA, USA). Extracted DNA was stored at −20 °C until analysis. Prior to analysis the DNA was split into two fractions, with one used for the analysis of prokaryotic and eukaryotic microbial diversity using (16S and 18S rRNA) amplicon sequencing, and the second fraction used for the quantification of DMSP degrading genes using real time quantitative Polymerase Chain Reaction (qPCR) described below.

For 16S amplicon sequencing, 20 µL aliquots of ≥10 ng/µL DNA were used and amplified using the 341F (CCTAYGGGRBGCASCAG) and 806R (GGACTACNNGGGTATCTAAT) primers and subsequently sequenced using the Illumina MiSeq platform (Illumina, San Diego, CA, USA) (Australian Genome Research Facility, Saint Lucia, VIC, Australia) following the manufacturer’s guidelines. The V4 Primers (>TAReuk454FWD1: CCAGCASCYGCGGTAATTCC; >V4_rev_Piredda: ACTTTCGTTCTTGATYRATGA) (Nextera-tagged primers) were used for 18S rRNA, with a thermocycling profile starting at 94 °C for 3 min; then 30 cycles of: 30 s at 94 °C, 60 s at 57 °C, 90 s at 72 °C; and a final 10 min at 72 °C. Amplicons were subsequently sequenced using the Illumina MiSeq platform (Illumina, San Diego, CA, USA) (Australian Genome Research Facility, Saint Lucia, VIC, Australia).

Gene sequencing reads were processed to an abundance table of Amplicon Sequence Variants (ASVs) from demultiplexed paired fastq files using DaDa2 (version 1.14.1 [56]). Briefly, after visual inspection of the read qualities the primers were removed from the forward and reverse reads using Cutadapt ([57]). Sequences were then filtered for N and trimmed with the following specific parameters Bacterial 16S: ‘truncLen = c(255,250), maxN = 0, maxEE = c(2,6), truncQ = 6, rm.phix = TRUE’. These parameters were identical for Eukaryote 18S with the exception of truncLen = c(200,195). Error rates were estimated from 1e8 bases with the option ‘max consist = 20’. Reads were then dereplicated and merged using the pool = “pseudo” option and potential chimeras were then removed using ‘removeBimeraDenovo’ with ‘minFoldParentOverAbundance’ = 4. The ASVs in the resulting abundance table were assigned to taxonomic lineages using ‘assignTaxonomy’ using Silva SSU taxonomic training data formatted for DADA2 (DOI: 10.5281/zenodo.3986799; Silva version 138) for 16S and PR2 version 4.13.0 for 18S (https://zenodo.org/record/3362765#.YL2O9y0RrUI, accessed on 19 April 2021). The dataset was further cleaned by removing sequence variants (ASVs) identified as chloroplasts, mitochondria and ASVs (abundance cut-off of 0.03%). Sequences were then rarefied to the same depth to remove the effect of sampling effort upon analysis. Raw FASTQ data files for the 16S rRNA and 18S rRNA assay were deposited in NCBI Sequence Read Archive (SRA) under Bioproject number PRJNA755150.

### 2.8. DMSP Degradation Gene Profiling

Total abundance of bacterial genes representing DMSP demethylation (DmdA subclade A1 and DmdA/Dall) and DMSP cleavage (DddP) were enumerated using quantitative polymerase chain reaction (qPCR; Biorad CFX384 real time PCR system) with the Biorad CFX384 manager software (Biorad, Hercules, CA, USA). Samples were run in technical triplicate of total reaction volume 5 µL containing; 2.5 µL of 2 × Sensifast Hi-rox master mix (Bioline, London, UK), 0.2 µL of each forward and reverse primer and 2.1 µL of DNA template). Thermocycling conditions of DmdA (A1_spF 5′-ATGGTGATTTGCTTCAGTTTCT-3′, A1_spR 5′-CCCTGCTTTGACCAACC-3′), (D/all_spF 5′-TATTGGTATAGCTATGAT-3′ and D/all-spR 5′-TAAATAAAAGGTAAATCGC-3′) and Ddd (dddP_874F 5′-AAYGAAATWGTTGCCTTTGA-3′, dddP_971R 5′-GCATDGCRTAAATCATATC-3′) involved a 5 min denaturation step at 95 °C, followed by 40 annealing temperature cycles of 54 °C, 42 °C and 41 °C, respectively (Supplementary Table S1 [58,59,60]) and a 30 s extension at 72 °C. All DMSP-related qPCR were 3 step assays. All primer specificity was verified using melt curve analysis. Gene abundances were quantified using a standard curve between 10^1^ copies L^−1^ and 10^6^ copies L^−1^ of prepared cloned gene fragments from environmental samples. Working stock solutions of a concentration of 10^10^ copies L^−1^ of each gene were prepared from purified gene fragments using a Qubit fluorometer (Invitrogen, Waltham, MA, USA). All DMSP degradation genes were normalised to 16S gene copy number (Supplementary Table S1 [60]).

### 2.9. Data Analysis

Differences in microbial abundances from flow cytometry and particulate DMSP for each size fraction were analysed by two-factor ANOVA, with site and experiment (microbial abundance) or site and treatment (DMPSp), as fixed factors. A Tukey’s post hoc test was performed to locate differences and account for multiple comparisons. Using a resemblance matrix based on Euclidean distance, a two factor Permutational Multivariate Analysis of Variance (PERMANOVA) with pair-wise comparisons was used to determine significant changes or differences in DMSPt and DMSPd concentrations over time and between treatments and to analyse the differences between treatments over time for the relative abundance of dmdA/A1, dmdA/Dall and DddP genes data. Differences in bacterioplankton (16S) and phytoplankton (18S) community composition between treatments over time (exp. 2) were analysed using PRIMER v6 statistical package [61]. Data were square root transformed and a resemblance matrix generated using Bray–Curtis similarity. Data were analysed using non-parametric Analysis of Similarities (ANOSIM) and discriminatory microbes identified using a two-way nested (time within treatment) Analysis of Similarity Percentages (SIMPER). Relationships between relative abundance of DMSP degrading genes and bacterial abundance (including all time points—T0, T24, T72 and T120 h and treatments (controls and DMSP-enriched)) were analysed using linear regression. All data analyses were performed in R 4.0.4 [62], using packages tidyverse [63], vegan [64] and ggpubr [65].

## 3. Results

### 3.1. Characteristics of Initial Water Masses

The physicochemical characteristics of the initial GBR water masses at the time of sampling were similar across both reef sites and experiments (Table 1). Initial water temperatures ranged between 26.3 and 27.1 °C for the two sites, while salinity did not vary (~35.4). Dimethyl sulfide (DMS) concentrations were ~0.9 nM at both sites, except for the inner reef site for experiment 1, where it was below the detection limit. Nutrient concentrations were generally very low, with nitrates (NO_X_) consistently low (≤0.02 µM) at both sites and for both incubation experiments. Phosphate concentrations were similar at both sites, ranging from 0.038 to 0.050 µM in the inner reef, and 0.049 to 0.066 µM in the outer reef, while silicate concentrations were 0.798 and 0.882 µM in the outer reef compared with 0.500 and 0.623 µM at the inner reef site for the two incubation experiments (Table 1).

Picoplankton abundances varied between sites and experiments, with generally higher abundances at the inner reef site (Table 2). There were higher abundances (F = 13.6, *p* = 0.006) of *Synechococcus* inside the reef compared with outside the reef (differing by an order of magnitude), while *Prochlorococcus* abundances were highest for experiment 1 (F = 23.54, *p* = 0.001) at the inner reef site (6.56 ± 2.7 × 10^5^ cell mL^−1^) and experiment 2 (5.59 ± 0.53 × 10^5^ cell mL^−1^) at the outer reef site. Heterotrophic bacterial abundances were higher at the inner reef site (0.79 ± 0.16 × 10^5^ cell mL^−1^) compared to the outer reef site (0.30 ± 0.05 × 10^5^ cell mL^−1^), with differences in abundance between experiments for the inner reef site (F = 25.8, *p* < 0.0001) and Picoeukaryote abundance was more than double inside (1490 ± 250 cell mL^−1^) than outside the reef (F = 37.8, *p* = 0.0002).

### 3.2. Experiment 1: Quantification of DMSP Uptake by Size Fractions of the Microbial Community

**Figure 2:**
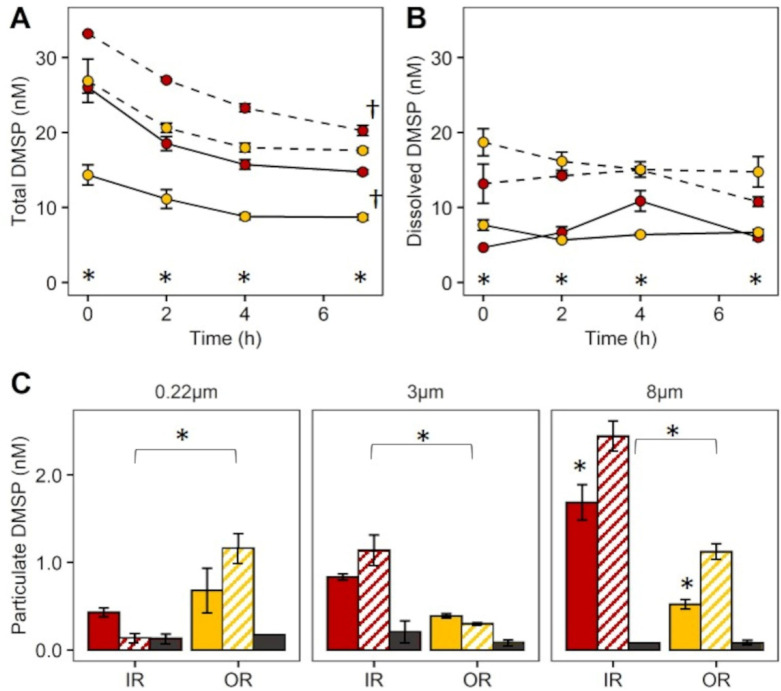
Concentrations of DMSPt and DMSPd over 7 h during and DMSPp in size fractionated community at 7 h. (**A**) Time course of DMSPt (**B**) Time course of DMSPd (**C**) DMSPp retained on 0.2 µm, 3 µm and 8 µm filters after 7 h, experiment 1. Inner reef site (Red) and outer reef site (Yellow). Control (solid line and colour), +DMSP (stippled line and striped colour), dark grey bars represent fixed (dead) samples. Data represent the mean ± standard deviation (*n* = 3). (**A**,**B**), significant change over time (†) and difference between treatment at each time point (*) at *p* < 0.05; (**C**), significance between site (indicated by horizontal bar and asterisk) and treatment (*) at *p* < 0.05.

Enrichment with DMSP resulted in significantly higher DMSPt concentrations for both the inner (F = 331, *p* = 0.001) and outer reef (F = 217, *p* = 0.001) incubations (Figure 2). During the 7 h incubation, the concentration of DMSPt at the inner reef site declined significantly from 26.1 to 14.8 nM in the control and from 33.2 to 20.3 nM in the DMSP-enriched samples (F = 99, *p* = 0.001), representing a loss of initial DMSPt of ~43% in both treatments (Figure 2A). While for the outer reef site, total DMSP concentrations remained relatively stable (12.3–8.70 nM) in the control and DMSP-enriched (22.6–17.6 nM) samples over 7 h (Figure 2A).

As expected, DMSP enrichment was immediately evident in the dissolved fraction, being significantly higher in the +DMSP treatment compared to the controls for both the inner (F = 37.4, *p* = 0.001) and outer (F = 217, *p* = 0.001) reef sites over time (Figure 2B). For the inner reef site, concentrations of DMSPd were 4.7 and 15.6 nM in the control and +DMSP samples, respectively, and for the control, DMSPd concentrations at the final time point were only marginally higher (6 nM) than at the outset (T0 h)—the abnormally high value of 10.88 nM at 4 h was likely due to filtration artifacts. For the outer reef site, DMSP remained constant in both the control and +DMSP treatments over time. There was no change in DMSPt and DMSPd concentrations over time for the fixed samples (data not shown).

Particulate DMSP levels varied according to size fraction and treatment (Figure 2C). Within Inner reef samples, the largest size fraction (>8 µm) contained the highest DMSPp concentrations (2.1 ± 0.5 nM), followed by the medium fraction (>3 µm; 0.99 ± 0.26 nM), and then the picoplankton (<3 µm; 0.28 ± 0.18 nM) fraction (Figure 2C). In contrast, DMSPp levels in the outer reef samples did not differ significantly across all three fractions in the absence of enrichment, but addition of DMSP led to significant increases (F = 80.43, *p* = 0.001) in DMSPp in the largest (>8 µm) fraction (Figure 2C). For the smallest fraction (0.22–3.0 µm), DMSPp levels were significantly higher in the outer reef community compared to the inner reef (F = 16.9, *p* = 0.0026), but with no effect of DMSP enrichment. In the 3–8 µm fraction, DMSPp levels were higher in the inner reef (F = 20.2, *p* = 0.0015), but enrichment did not result in a further increase in DMSPp. For the largest fraction (>8 µm), the inner reef had higher levels of DMSPp, and enrichment increased DMSPp at both sites (F = 8.47, *p* = 0.008). There was no change in DMSPp in fixed cells with DMSP enrichment, which remained low in all fractions (Figure 2C).

**Figure 3:**
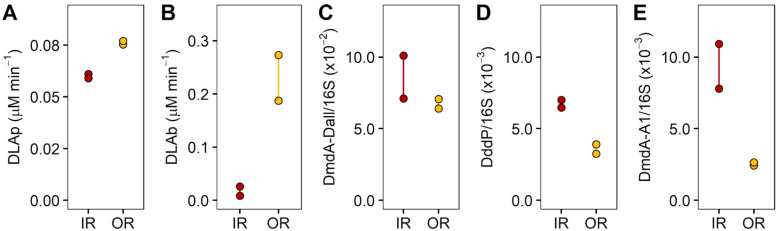
DMSP lyase activity (DLA) rates for phytoplankton and bacterial fractions and relative abundance of DMSP degradation genes. Rates of DLA for (**A**) phytoplankton (DLAp), (**B**) bacteria (DLAb), µM DMS min^−1^. Proportion of (**C**) DmdA/Dall, (**D**) DddP and (**E**) DmdA/A1 normalised to 16S copy abundance for initial waters for inside the reef site (IR) and outside the reef site (OR) for experiment 1. Data show duplicate measurements (*n* = 2).

Rates of DMSP lyase activity (DLA) differed between sites (Figure 3). The inner reef was dominated by phytoplankton DLA (DLAp), with rates three-times higher (0.06 µM DMS min^−1^) than those of the bacterial (DLAb) fraction (0.017 µM DMS min^−1^; Figure 3A). In contrast, a reverse pattern was found for the outer reef site, where DLAb rates (0.23 µM DMS min^−1^) were three times higher than DLAp rates (0.07 µM DMS min^−1^; Figure 3B). Interestingly, DLAb rates in the outer reef were more than an order of magnitude higher than those measured for the inner reef, while DLAp rates were similar across both sites. Of the DMSP catabolising genes tested, DmdA/Dall occurred in the highest abundance at both sites, with 7–10 × 10^−2^ copies per 16S copies for the inner reef site and 6.5–7 × 10^−2^ copies per 16S copies for the outer reef site (Figure 3C). The common lyase gene DddP was approximately twice as abundant (6.7 × 10^−3^ copies/16S) in the bacterial population of the inner reef compared with the outer reef site (3.6 × 10^−3^ copies/16S; Figure 3D). As with DddP, the abundance of the gene DmdA/A1 was more abundant in the bacterial populations of the inner reef (9.4 × 10^−3^ copies/16S) than the outer reef site (2.5 × 10^−3^ copies/16S; Figure 3E).

**Figure 4:**
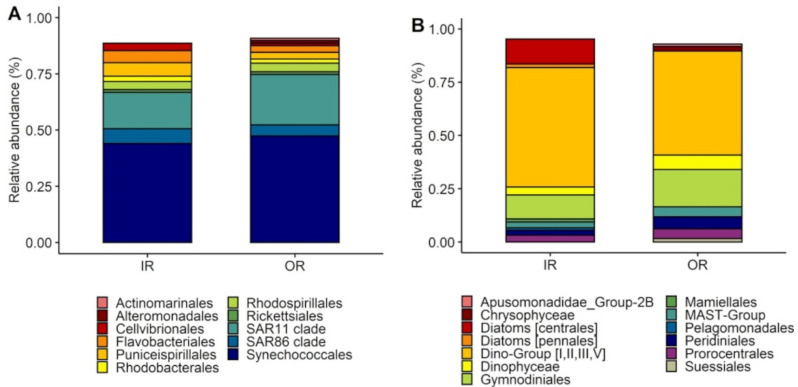
Bacterial and phytoplankton composition. Community (16S and 18S) composition of initial waters for both sites for experiment 1. Relative abundance of (**A**) bacteria and (**B**) phytoplankton for inner (IR) and outer (OR) reef sites. Graphs display orders with >1% of relative abundance for clarity purposes (*n* = 1).

Bacterioplankton community composition was similar at both sites, with up to eleven genera of >1% of relative abundance making up more than 80% of the community (Figure 4A). Reef waters were dominated by Synechococcales (44–47%) and SAR11 clade (16–22%). The remaining genera present in >1% relative included abundance consisted of genera from the orders of SAR86, Puniceispirillales, Flavobacteriales, Rhodospirillales, Cellvibrionales, Rhodobacterales, Rickettsiales, Alteromonadales and Actinomarinales (1.03%). At both sites, the dominant genus within the Synechococcales order was the photosynthetic cyanobacterium from the genus *Prochlorococcus*, while the other important photosynthetic cyanobacterium *Synechococcus* was present at 3.6% in the inner reef site, yet <1% at the outer reef. Phytoplankton community composition was similar between sites (Figure 4B) with dinoflagellates (Dino-Group I–V, 48–56%; Gymnodiniales, 11–17%; Dinophyceae, 3.7–6.8%; Prorocentrales, 3–4.6%; Peridiniales 2–5.6%) as the dominant algal class (65% IR, 85% OR) at both sites. The only notable difference in community composition was the presence of diatoms at the inner reef site, which had a relative abundance of 13%, and were completely absent from the outer reef site.

### 3.3. Experiment 2: Effect of DMSP on Community Structure and Gene Regulation

To evaluate the effect of DMSP-enrichment on microbial community composition and DMSP catabolising gene abundance in natural seawaters of the GBR, we incubated water samples with trace levels of DMSP (final concentration 10 nM) and monitored changes in microbial communities and sulfur compounds over five days. Macronutrient concentrations over time differed between the inner and outer reef experiments, where the inner reef, phosphate dropped below detection limit by 120 h, and silicate concentrations increased from initial concentrations (Supplementary Table S2). For the outer reef silicate also increased by 120 h, but there was no change in phosphate (Supplementary Table S3). Concentrations of NO_X_ and ammonium remained low throughout.

**Figure 5:**
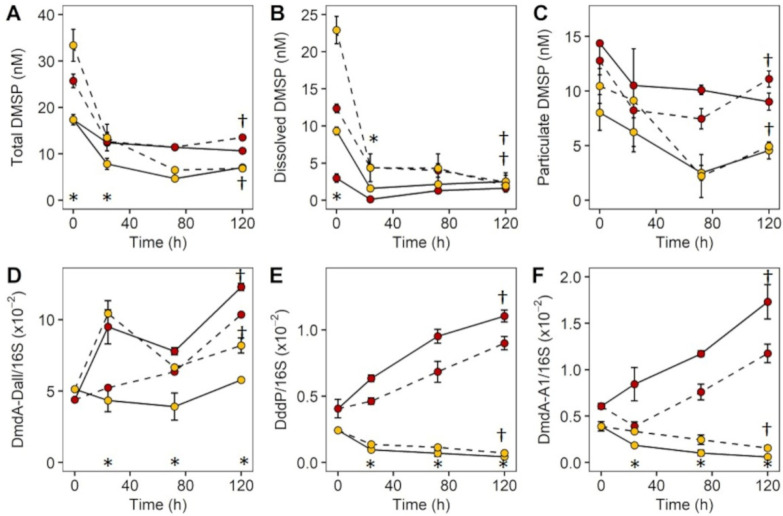
Concentrations of DMSPt, DMSPd, DMSPp and DMSP degradation genes over 120 h. Time course of (**A**) DMSPt, (**B**) DMSPd, (**C**) DMSPp, DMSP degradation genes, (**D**) DmdA/Dall, (**E**) DddP, (**F**) DmdA/A1 (copies/16S copies) for the inner reef (Red) and outer reef (Yellow) sites. Control (solid line and colour), +DMSP (stippled line and striped colour). Data represent the mean ± standard deviation (*n* = 3). Significant change over time (†) and difference between treatment at each time point (*) at *p* < 0.05.

For the inner reef site, DMSPt declined significantly (F = 35.2, *p* = 0.001) from 17.36 to 10.64 nM in the control and 25.72 to 13.54 nM in the DMSP-enriched incubations (Figure 5A). Most of the decline in DMSPt occurred within the first 24 h, with a disappearance rate of 5.04 nM day^−1^ for the controls and 12.24 nM day^−1^ for DMSP-enriched samples, equating to almost half of the total DMSP, and treatment differences (F = 11.1, *p* = 0.013) disappeared after 24 h. Outside the reef, DMSPt declined (F = 67.7, *p* = 0.001) from 17.35 to 7.08 nM in the control and from 33.38 to 6.9 nM in the DMSP-enriched samples (Figure 5A). As with the inner reef, the greatest decline occurred during the first 24 h, with treatment differences (F = 20.06, *p* = 0.005) disappearing after 24 h, but at a higher loss rate, 9.36 nM day^−1^ for the controls and 19.92 nM day^−1^ for the DMSP-enriched samples.

For the inner reef, concentrations of DMSPd remained constant at ~2 nM in the control over time, while DMSP-enriched samples showed a rapid and significant decline in the first 24 h (F = 38.8, *p* = 0.001), followed by a steady, slow continuous decrease over the remaining 96 h (Figure 5B). For the +DMSP samples, a total of 80 % of initial DMSPd was lost in 120 h, with a loss rate of 8.64 nM day^−1^ for the first 24 h and 0.47 nM day^−1^ for the next 96 h. A different pattern was detected for outer reef samples, where DMSPd declined significantly (F = 67.05, *p* = 0.002) from 9.33 to 2.50 in the controls and from 22.91 to 1.94 nM in the DMSP-enriched samples (Figure 5B). Like the inner reef, the highest rate of loss occurred during the first 24 h with 7.74 nM day^−1^ (controls) and 18.56 nM day^−1^ (+DMSP). This loss of DMSPd over 120 h equates to a loss of the initial DMSPd of approximately 73% in the controls and ~91% in DMSP-enriched bottles. As with DMSPt, the rapid decline meant that initial differences in DMSPd between treatments for the inner (F = 71.8, *p* = 0.001) and outer (F = 21.3, *p* = 0.006) reefs disappeared after only 24 h.

Particulate DMSP for the inner reef samples, calculated by subtracting DMSPd from DMSPt (Figure 5C) remained relatively constant (~12 nM) for the DMSP-enriched samples, while declining significantly from 14 nM to 9 nM in the controls (F = 7.19, *p* = 0.015) with a loss rate of 1.07 nM day^−1^. Conversely, DMSPp remained constant at ~6 nM for the controls yet significantly diminished from 10.46 nM to 4.94 nM for the DMSP-amended samples (F = 5.01, *p* = 0.020) at a rate of 1.10 nM day^−1^ in the outer reef samples (Figure 5C).

Contrasting trends in DMSP degradation gene abundance were evident between the two sites, with significant increases over time for the inner reef community, compared with a stable or declining gene abundance over time for the outer reef community (Figure 5D–F). Inside the reef, catabolising genes DmdA/Dall (F = 81.8, *p* = 0.001), DddP (F = 37.2, *p* = 0.001) and DmdA/A1 (F = 35.6, *p* = 0.001) increased over the 120 h, with faster rates of increase (DallF = 36.4, *p* = 0.002; DddP F = 10.2, *p* = 0.011; A1F = 23.1, *p* = 0.002) in the control compared with DMSP-enriched samples (Figure 5D–F). The inverse pattern was observed outside the reef, where the higher relative abundances of all three genes were in the +DMSP samples and DddP (F = 143.4, *p* = 0.001) and DmdA/A1 (F = 18.4, *p* = 0.001) decreased significantly over 120 h (Figure 5E,F). Despite an apparent spike in DmdA/Dall at 24 h for the DMSP-enriched population, gene abundance increased over the 120 h (F = 10.5, *p* = 0.003) and maintained a higher relative abundance (F = 49.4, *p* = 0.001) than the controls (Figure 5D). DMSP lyase activity for the phytoplankton (DLAp) and bacterial (DLAb) fractions were similar (between 0.47–0.79 µM DMS min^−1^) for the inner reef site. In contrast, DLAp rates were approximately four times higher than rates of DLAb for the outer reef site (0.04 and 0.01 µM DMS min^−1^, respectively), and were an order of magnitude lower than those measured for the inner reef community (data not shown).

**Figure 6:**
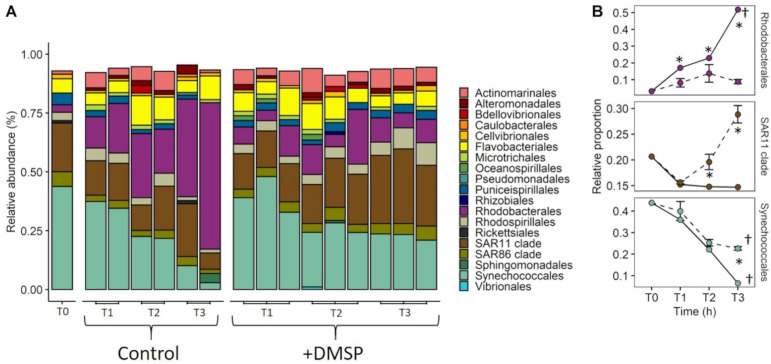
Inner reef bacterial community composition, experiment 2. Relative abundance of (**A**) bacterial orders that make up >1% of the total community, at T0, 24 (T1), 72 (T2) and 120 h (T3) for control (*n* = 2) and +DMSP (*n* = 3) incubations from the inner reef site. (**B**) Change in the relative proportion of Rhodobacterales, SAR11 clade and Synechoccocales over time for control (solid lines) and +DMSP (dashed lines) samples from the inner reef. Data are based on prokaryotic (16S) sequences. (**B**): Data represent the mean ± standard deviation (*n* = 3). Significance over time (†) and between treatment at each time point (*) at *p* < 0.05.

As with the first experiment, the bacterial community from the inner reef site for experiment 2 was dominated by genera from the orders Synechococcales (44%; *Prochlorococcus* 34% and *Synechococcus* 10%) and SAR11 clade (20%), together making up 64% of the community (Figure 6A). Other genera at >1% relative abundance included SAR86, Flavobacteriales, Puniceispirillales, Rhodospirillales, Rhodobacterales, Cellvibrionales, Actinomarinales and Rickettsiales. Community structure changed significantly over time (120 h) and between treatments (ANOSIM; global R = 0.477, *p* = 0.002), with Rhodobacterales, Synechococcales, Actinomarinales, SAR11 clade and Rhodospirillales forming the major groups contributing to 54.6% of the cumulative difference (Supplementary Table S4). There was an increase in overall diversity over time, with the appearance of several new orders (Alteromonadales, Caulobacterales, Chitinophagales, Microtrichales, Pseudomonadales, Sphingomonadales, Alteromonadales and Microtrichales) >1% relative abundance. The key changes across treatments from the starting community were the dramatic increase in the relative abundance of Rhodobacterales (predominantly from the genus *Sulfitobacter*), which reached 50% in the controls, compared with only 8% in the DMSP-enriched samples (F = 6.8, *p* = 0.025; Figure 6B). While controls saw a decline in the relative abundance of SAR11 clade, DMSP-enriched communities exhibited an increase in the relative abundances, making up ~28% of the final community (F = 5.9, *p* = 0.038). In both treatments we saw a decline in relative abundance of Synechococcales after 120 h (F = 33.9, *p* = 0.001), where final abundances were lower in the controls (<10%) compared with the +DMSP (~28%) treatment (F = 8.3, *p* = 0.018; Figure 6B).

**Figure 7:**
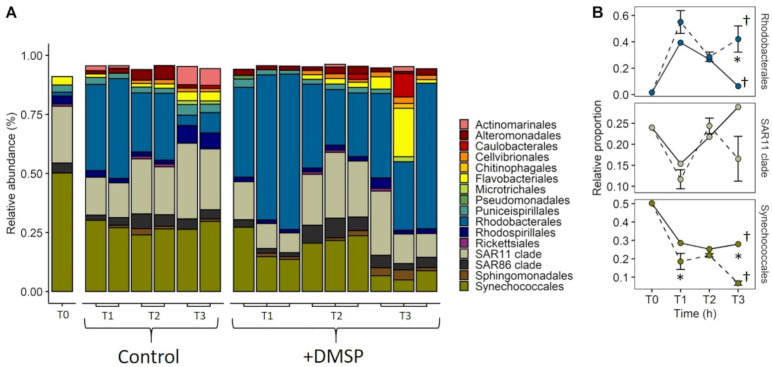
Outer reef bacterial community composition, experiment 2. Relative abundance of (**A**) bacterial orders that make up >1% of the total community, at T0, 24 (T1), 72 (T2) and 120 h (T3) for control (*n* = 2) and +DMSP (*n* = 3) incubations from the outer reef site. (**B**) Change in the relative proportion of Rhodobacterales, SAR11 clade and Synechoccocales over time for control (solid lines) and +DMSP (dashed lines) samples from the outer reef. Data are based on prokaryotic (16S) sequences. (**B**): Data represent the mean ± standard deviation (*n* = 3). Significance over time (†) and between treatment at each time point (*) at *p* < 0.05.

For the outer reef site, the prokaryotic community resembled the initial inner reef waters (Figure 7A), with strong changes in community structure over 120 h that differed between treatments (ANOSIM; global R = 0.617, *p* = 0.001). For the outer reef, the major groups contributing to 53.61% of the cumulative difference were Rhodobacterales, Synechococcales, Actinomarinales and Sphingomonadales (Supplementary Table S4). As with the inner reef, both treatments saw an increase in diversity, with several new orders (Alteromonadales, Caulobacterales, Chitinophagales, Microtrichales, Pseudomonadales and Sphingomonadales) appearing at >1%. Of the initial community, we saw a strong initial (24 h) increase in the relative abundance of Rhodobacterales in both treatments (F = 8.03, *p* = 0.008). In the +DMSP treatment this shift towards Rhodobacterales, (predominantly *Sulfitobacter*) was retained for 120 h, becoming the dominant order (~42%) within the community at 120 h, whereas in the control, the relative abundance returned to low numbers (~6%) by the end of the experiment (Figure 7B). In both treatments, SAR11 clade showed no change in abundance through time, while the relative abundance of Synechococcales declined in both treatments (F = 39.1, *p* = 0.001), but remained higher (F = 35.3, *p* = 0.001) in the control (~27%) than the +DMSP (~7%) treatment (Figure 7B).

**Figure 8:**
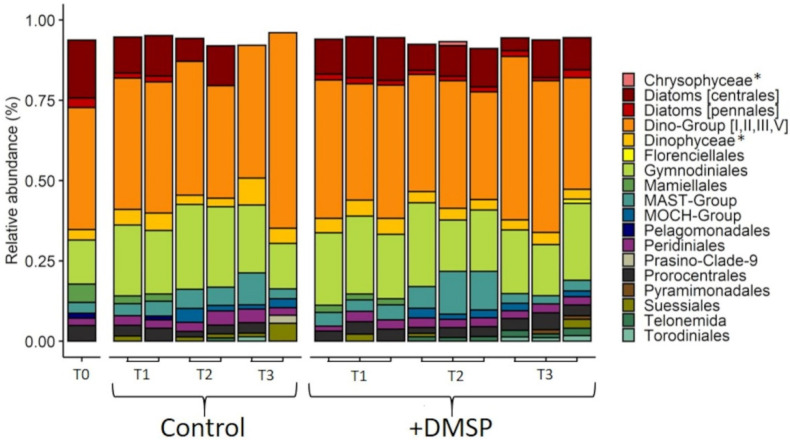
Taxonomic composition of phytoplankton from the inner reef community, experiment 2. Relative abundance of phytoplankton taxa that make up >1% of the total community at T0, 24 (T1), 72 (T2) and 120 h (T3) for control and +DMSP incubations from the inner reef site. Data are based on eukaryotic (18S) sequences. * In cases where taxonomic identification could not be made to lower level than class, class is shown.

Phytoplankton community composition of the inner reef was dominated by dinoflagellates belonging to seven different taxonomic groupings (Figure 8). Dino-Group (I, II, III and V) made up the highest relative abundance (38%), followed by diatoms from the order Centrales (18%). Dinoflagellates from the order Gymnodinales (13%) had the next highest relative abundance within the initial inner reef phytoplankton community. The remaining phytoplankton taxa consisted of chlorophytes from the order Mamiellales (5.6%), dinoflagellates from the orders Prorocentrales (4.8%) and Peridiniales (2.3%), uncultivated marine Stramenopiles—MAST-group (3.4%), pennate diatoms (2.9%) and Pelagomonodales (1.5%). Changes in community structure were observed for both the control and DMSP-enriched communities over the 120 h (ANOSIM; global R = 0.556, *p* = 0.002) with five orders—Diatoms (centrales), Dino-Group (I, II, III, V), Gymnodiniales, Suessinales and Diatom (pennales)—contributing to 51.41% of the dissimilarity between treatments (Supplementary Table S4).

**Figure 9:**
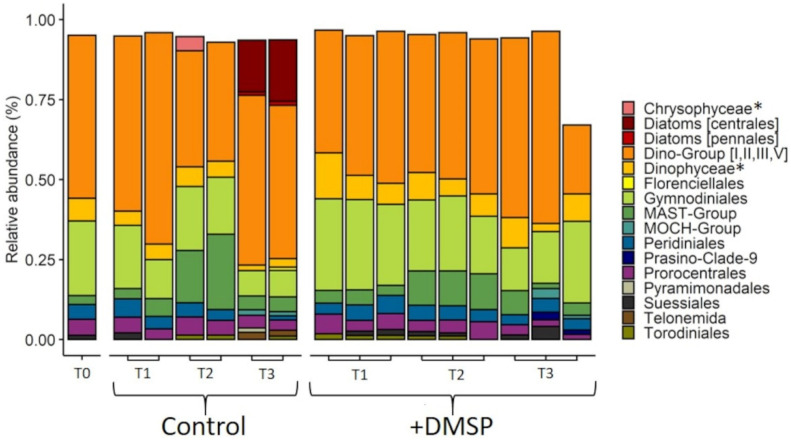
Taxonomic composition of phytoplankton from the outer reef community, experiment 2. Relative abundance of phytoplankton taxa that make up >1% of the total community at T0, 24 (T1), 72 (T2) and 120 h (T3) for control and +DMSP incubations from the outer reef site. Data are based on eukaryotic (18S) sequences. * In cases where taxonomic identification could not be made to lower level than class, class is shown.

For the outer reef, the phytoplankton community structure strongly resembled that of the inner reef, with a dominance of dinoflagellates, but a notable absence (<1%) of diatoms (Figure 9). As with the inner reef, Dino-Group (I, II, III and V) made up the highest relative abundance (51%), followed by dinoflagellates from the Gymnodinales (23%), Dinophyceae (7%), Prorocentrales (5%), Peridinales (4.6%) and Suessiales (1.3%). The only non-dinoflagellate taxa present at >1% in the initial community belong to the MAST-Group (2.7%). As with the inner reef community, there were shifts in community structure over 120 h (ANOSIM; global R = 0.521, *p* = 0.001), with Dino-Group (I, II, III, V), Diatoms (centrales), MAST-Group, Gymnodiniales and Stramenopiles as the major contributors to 53.49% of the dissimilarity between treatments (Supplementary Table S4).

**Figure 10:**
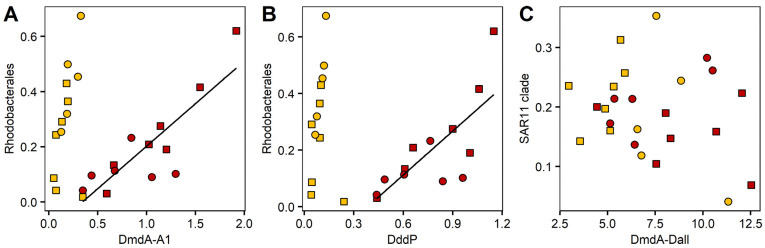
Regression analysis of the relative abundance of DMSP-degrading genes and bacterial abundances of target groups for inner (red) and outer (yellow) reef sites. The relative abundance of (**A**) Rhodobacterales and DmdA/A1, (**B**) Rhodobacterales and DddP, (**C**) SAR11 and DddP. DMSP degradation genes are normalised to 16S copy numbers. Squares, controls; circles, +DMSP. Lines show significant linear regressions (*p* < 0.05).

To test for relationships between DMSP catabolising taxa and the relative gene abundance, linear regressions were performed on selected prokaryotic orders known to harbour relevant taxa (Figure 10). Specifically, qPCR primer target groups SAR11 (DmdA/Dall; [66]) and Rhodobaterales, which harbour Roseobacters, the target group for A1 and DddP primers [58,66] were tested. We found significant relationships between Rhodobacterales and both 16S normalised DmdA/A1 (adjR^2^ = 0.67; *p* = 0.0003) and DddP (adjR^2^ = 0.52; *p* = 0.002), but only for the inner reef site (Figure 10A,B). This relationship appeared to be driven by the abundance of the Roseobacter genus *Sulfitobacter* (Supplementary Figure S1), where the significant relationships explained 67% (DmdA/A1) and 52% (DddP) of the variation. We found no relationships between the relative abundance of SAR11 clade and DmdA/Dall at either reef site (Figure 10C).

## 4. Discussion

Corals and reef lagoons of the GBR are well characterised as DMS/P hot spots, due to high local production by corals [47]; however, limited information exists on DMSP concentration and cycling outside shallow reef waters. Dimethylsulfoniopropionate (DMSP), which in the open ocean is predominantly produced by marine phytoplankton and taken up by several groups of marine heterotrophic bacteria [6,7] plays an important role in marine ecosystems. While there is considerable evidence for bacterial uptake and processing of DMSP [29,66], little research exists on the uptake of DMSP by non-DMSP producing phytoplankton [33,34]. Using a combination of short- and longer-term DMSP enrichment experiments, we investigated which members of the marine microbial community take up available DMSP from the environment and the effect that increasing DMSP availability has on the abundance of DMSP degradation genes and microbial community composition in natural sea waters of the Great Barrier Reef, Australia.

### 4.1. Quantification of DMSP Uptake by Size Fractions of the Microbial Community—Experiment 1

The seawater characteristics of both sites were typical of coral reef waters, with nutrients and temperature values within ranges of previous studies made on the GBR [67,68,69]. Measured DMS/P concentrations from both reef sites fell within the range previously found in the GBR, albeit at the lower end. Previous studies have measured DMS concentrations of ~2.0 (range; BDL-54) nM and ~1.3 (BDL-3.9) nM in the reefs of Orpheus and Magnetic islands, respectively [48], and DMSP values of ~3.8 (0.36–35) nM at One Tree Reef [46]. The relatively low values of DMS/P measured in this study are likely due to the location of the sampling points and time of collection; as peak DMS concentrations have been measured in high summer above the reef flat [48], whereas our data were sampled in October and collected from open water ~13 km from the closest reef.

The patterns of DMSP uptake by the different fractions of the marine microbial communities of the GBR determined over 7 h (experiment 1) showed a loss of DMSPt, for both sites and treatments, indicating that DMSP was escaping from the system. This could occur via bacterial conversion to DMS, DMSO and MeSH or by photochemical oxidation to DMSO [70,71,72]. As DMSPp accumulated with enrichment, we suggest that a significant fraction of available DMSPd was taken up and transformed, either to satisfy the sulfur demand in form of MeSH, to cleave into DMS and be lost to the atmosphere, or to oxidise to DMSO to protect the cells from ROS [10,21,37]. At both reef sites, DMSP enrichment led to greater DMSP uptake in the largest (8 µm) fraction, supporting the findings that phytoplankton, as well as bacteria take up DMSP [18,34,35,73], providing new evidence that the larger members of the microbial community can act as important DMSP sinks in coral reef waters. The differences in DMSP uptake between sites may be due to differences in the eukaryotic composition, where the presence of diatoms at the inner reef site may account for the greater uptake with DMSP enrichment [34].

At both reef sites, prokaryotic communities were dominated by the photosynthetic cyanobacteria *Prochlorococcus*, typical of warm oligotrophic areas, due to its adaptability to thrive in nutrient poor waters [74,75]. Because of its high abundance and known ability to take up DMSP [33,76], it is possible that this taxon plays an important role in DMSP cycling on the reef and may be an important DMSP sink within the smallest size fraction (>0.22 µm fraction). While previous work has shown that cyanobacteria take up DMSP, little is known whether they catabolise it. To date, only one sequenced cyanobacterial strain, *Synechococcus* sp. KORDI-100, has been found to have a DMSP lyase gene [77]. Given that *Prochlorococcus* and SAR11 were present in high relative abundances at both sites, the fact that DMSP showed higher accumulated in the small fraction of the outer reef, suggests that rather than differences in microbial community composition, it was the metabolic state of the community driving this difference, whereby the bacterial community of the outer reef site were preferentially taking up DMSPd to meet their sulfur and carbon demands for growth [21,27]. Given the difference in depth of the two sites and therefore potentially different mixing regimes, the influence of mechanistic controls, such as UV-A dose [58], on the bacterial switch cannot be ruled out as a potential driver of the differences between sulfur demand for the two sites.

We found dinoflagellates dominated the reef waters from both sites (Figure 4). Given that dinoflagellates are known to be some of the highest-DMSP producers [2,5], it is likely that species from this class constituted the primary source of DMSP in these waters. The higher concentrations of DMSP inside the reef, suggests a greater absolute abundance of these cells, with the possibility of additional contributions from dinoflagellate symbionts associated with corals [46,47,48]. In support of higher biomass, the fluorescence trace from the CTD revealed the chlorophyll *a* signature was twice as high at the inner reef site compared with that of the outer reef, indicating higher phytoplankton biomass in the inner reef surface waters. As such, we propose that the dinoflagellates accounted for the high proportion of DMSPp in the >8 µm fraction, but that the main taxa responsible for the uptake of additional DMSP in the +DMSP treatment be attributed to the diatoms, which have been shown to take up and accumulated DMSP in high concentrations [34]. The absence of diatoms from the outer reef site, suggests that other large non-DMSP producing phytoplankton may act as DMSP sinks, but further work is needed to confirm uptake and identify these groups.

At both sites, the most abundant DMSP degrading pathway was demethylation, represented by the gene DmdA (Dall and A1). Similar relative abundances of these DMSP degradation genes using qPCR have been reported for the North Pacific subtropical gyre [78]. The DmdA gene is responsible for the first step of the demethylation pathway and so, it is likely that the disappearance of DMSP from the incubations over 7 h for both reef sites was through MeSH production [29]. Of the bacterioplankton, the groups most likely to demethylate DMSP in theses water are SAR11 and members of the Rhodobacterales [29]. However, as SAR11 were more abundant than Rhodobacterales at both reef sites, we speculate that they may form the dominant DMSP consumer in the surface waters of the GBR, a finding commensurate with those for the Sargasso Sea and North Atlantic Ocean [79], Tropical and Subtropical Pacific Ocean [80], and East China sea [81]. With respect to DMSP cleavage via DddP genes, these processes can be attributed to several genera from within the Rhodobacterales, many of which have been shown to have strong correlations with DMS production [81]. It is important to note however, that the highly conserved nucleotide sequences of the primers used in qPCR means that copy numbers recorded in this study likely underestimate total gene abundance in the GBR reef waters, as many sequences are left out of the analysis. For example, in the case of the demethylation gene DmdA, Dall primers target SAR11-like sequences, while A1 targets Roseobacter-like sequences [29], and while both clades D and A have been shown to make up the majority of sequences when a universal DmdA primer has been used [78], the primer sets used in this study do not cover all DmdA subclades. Similarly, it is impossible to rule out that some bacterioplankton lacking DMSP degradation genes may take up DMSP for other reasons, such as for osmolytic benefit, as shown in a previous study, in which coastal seawater filtrates containing mostly bacteria, diminished their MeSH production and retained up to 54% of their intracellular DMSP when under osmotic stress [82].

### 4.2. Dynamic Changes in Microbial Community Composition and DMSP Degradation Gene Abundance in Reef Waters—Experiment 2

Longer-term incubations designed to follow DMSP-induced community changes and shifts in catabolising gene abundance revealed strong differences between sites and treatments. For both sites, concentrations of DMSPt and DMSPd decreased over time in both control and DMSP-enriched treatments, with DMSP consumption rates (IR: 5–12, and OR 9–20 nM d^−1^) within previously reported values (7–80 nM d^−1^; [83,84]). These data indicate that DMSP was rapidly (within hours) taken up from the solution and then lost within 24 h via production of DMS or MeSH [21], in accordance with experiment 1. The lack of intracellular accumulation (increase in DMSPp) over several days suggests that the DMSP that was taken up within the first few hours and was being utilised or converted, regardless of DMSP availability, a finding that is in accordance with previous results where added ^35^S-DMSPd was transformed into several products over time scales of minutes to one day [66].

To date, DMSP lyase activity (DLA) in phytoplankton has only been shown in *Emiliania huxleyi* [85], *Phaeocystis* spp. [86] and different Symbiodiniaceae genera [87,88], all high DMSP producers. As such, we hypothesise that the DLAp measure in these GBR waters is likely attributable to the high DMSP-producing dinoflagellates that dominated the phytoplankton communities at both sites. For the bacterial fractions, the high rates of DLAb of the inner reef suggest that DMSP concentrations may have exceeded the necessary levels to cover the sulfur demands of the bacterioplankton community, and therefore much of the excess was cleaved to DMS [21,26,27]. In contrast, outside the reef, it is likely that most of the added DMSP was utilised to meet the sulfur demands of the bacterioplankton community. Taken together, our data suggest that the inner reef site had higher inputs of organic carbon and sulfur and more efficient nutrient recycling systems, while the outer reef site was more representative of the oligotrophic open ocean, whereby DMSP enrichment provided cells some relief from carbon and sulfur limitation.

As with the short-term experiments, DmdA/Dall was the dominant DMSP degradation gene throughout the experiment (120 h) with similar relative abundances at both sites at the outset when most of the DMSP was lost from the system, suggesting that most of the DMSP was degraded through demethylation [22,23]. These data are congruent with previous studies, where DmdA subclade D is present at an order of magnitude higher than DmdA/A1 and DddP [29,30,58,78,81]. The relative abundances of the different DMSP degradation genes over time showed variable behaviours for the two locations. In agreement with the DLAb data, all degradation genes increased in relative abundance with time at the inner reef site, suggesting that sulfur demands were met quickly [27], after which cleavage to DMS dominated. For the outer reef site, the relative abundance of genes was higher in the DMSP-amended samples compared to the controls, indicating that the microbial community from the outer reef was partially advantaged by DMSP enrichment, but overall abundances declined over time. These data are supported by the low DLA levels measured for this site, strengthening the idea that the DMSP concentrations in the oligotrophic waters outside the reef were insufficient to completely satisfy the sulfur and carbon demands of the outer reef microbial community.

In favouring DMSP utilisers, enrichment with DMSP can disrupt competitive hierarchies in microbial communities, allowing some taxa to proliferate over others. We saw DMSP-related changes in the phytoplankton community over the 120 h at both sites, predominantly between the diatom and dinoflagellate groups, indicating an interplay between DMSP sources and sinks within the phytoplankton community. For the prokaryotic community we measured key changes over time, some of which were in response to DMSP enrichment and could be linked with changes in gene abundance (Figure 10). In particular, the proliferation of SAR11 clade with DMSP addition at the inner reef site suggest that these taxa were able to monopolise on increased DMSP availability, despite finding no relationship with demethylation gene abundance. The absence of a significant relationship between SAR11 clade and DmdA/Dall, indicates that SAR11 clade may not be the major contributors to MeSH production via demethylation in GBR waters. It does not preclude the possibility, however, that SAR11 clade may have contributed to the high DLAb activity measured at the inner reef site, via the DddK gene [32,89], but this was not measured directly here. Contrastingly, Rhodobacterales, known to harbour several genera with the capacity to utilise DMSP [81], showed no benefit from DMSP enrichment at the inner reef site, yet exhibited strong correlations with DMSP catabolising gene abundances (both DmdA and DddP), suggesting that taxa within this order are the dominant DMSP degrading bacteria in these inner coral reef waters. Rhodobacterales have been previously found to positively correlate with relative abundances of both DddP and DmdA [80,81], and are one of the major groups responsible for DMSP demethylation in the marine environment [29]. In particular, *Sulfitobacter*, a genus within the Rhodobacter order and the dominant genus in our study, have been previously linked with these genes and shown to use DMSP as their sole carbon source for MESH production [81]. These observed differences in community responses to DMSP enrichment between the two sites are compatible with the theory of the bacterial switch, whereby enrichment of the inner reef community resulted in the sulfur demand being satisfied and the subsequent loss of DMSP from the system via cleavage to DMS, whereas for the more oligotrophic waters of the outer reef, even with DMSP enrichment, the DMSP availability was insufficient to completely satisfy the sulfur and carbon requirements of the community.

## 5. Conclusions

In this study, we evaluated the uptake of DMSP by different fractions of marine microbial communities of the GBR and revealed that both bacteria and phytoplankton from natural reef waters can take up DMSP over short time scales. Specifically, DMSP enrichment revealed the main sink for DMSP comprised of taxa from the largest microbial fraction. By investigating the main DMSP catabolising genes over 120 h, we were able to show that despite similarities in microbial community composition, strong differences in DMSP catabolism and thus influence on sulfur cycling are possible. We found that taxa from the Rhodobacterales order best explained the increases in DMSP catabolising genes, but this relationship was only found for the inner reef site. The differences in DMSP lyase activity, catabolism and community structure observed between the two reef sites suggests that at the inner reef site sulfur and carbon demands were largely satisfied by existing DMSP availability, meaning that DMSP addition had minimal impact on the microbial community from these waters, with lyase activity dominating the conversion of DMSP to DMS. On the other hand, the absence of a strong response to DMSP enrichment for the outer reef bacterioplankton community (minimal increases in abundance and no positive relationships with DMSP catabolising genes), when taken in the context of the low DLA rates measured, suggests minimal DMSP cleavage to DMS occurring, and therefore we may presume that any DMSP in the system was being converted to MeSH or lost from the system via oxidation. Overall, our findings contribute to an improved understanding of the fate and function of DMSP in tropical marine ecosystems and identify microbial DMSP sinks and sources within the deeper, oligotrophic waters of the GBR.

## Figures and Tables

**Table 1:** Characteristics of water masses for the inner and outer reef sites. The sampling depth, temperature, salinity and DMS concentration from the initial water sampled at both sites and experiments. Concentrations of nitrates (NO_X_), nitrite (NO_2_^−^), phosphate (PO_4_^3−^), silicate (SiO_4_^3−^) and ammonium (NH_4_^+^) at the initial sampling time point for the inner reef and outer reef sites for both experiments. Data obtained from a single measurement. BDL = below detection limit.

	Inner Reef		Outer Reef	
	Experiment 1	Experiment 2	Experiment 1	Experiment 2
Sample depth (m)	4.0	3.4	5.0	5.0
Temperature (°C)	26.3	27.1	26.7	26.5
Salinity (psu)	35.4	35.4	35.4	35.5
DMS (nM)	BDL	0.90	0.86	1.0
NO_X_ (µM)	0.014	0.020	0.020	BDL
NO_2_^−^ (µM)	0.006	0.007	0.004	0.020
PO_4_^3−^ (µM)	0.038	0.050	0.049	0.066
SiO_4_^−^ (µM)	0.623	0.500	0.798	0.882
NH_4_^+^ (µM)	BDL	0.010	0.021	0.004

**Table 2:** Microbial abundance (cells mL^−1^) measured using flow cytometry. Data represent mean ± SD (*n* = 3). Superscript letters denote statistical differences at *p* < 0.05 between sites and experiments. Unique lettering indicates significantly different from all other treatments, whereas shared superscript letters denote non-significance between treatments.

	Inner Reef		Outer Reef	
	Experiment 1	Experiment 2	Experiment 1	Experiment 2
*Synechococcus* (cells × 10^3^ mL^−1^)	2.65 ± 0.71 ^a^	1.73 ± 0.03 ^ac^	0.20 ± 0.14 ^b^	0.93 ± 0.02 ^bc^
*Prochlorococcus* (cells × 10^5^ mL^−1^)	6.56 ±2.7 ^ac^	2.11 ± 0.15 ^b^	2.29 ± 0.06 ^b^	5.59 ± 0.53 ^bc^
Heterotrophic Bacteria (cells × 10^5^ mL^−1^)	9.30 ± 0.05 ^a^	6.50 ± 0.02 ^b^	3.24 ± 0.02 ^c^	2.67 ± 0.05 ^c^
Picoeukaryotes (cells mL^−1^)	1566 ± 361 ^a^	1416 ± 57.0 ^a^	616 ± 246 ^b^	716 ± 144 ^b^

## Data Availability

Sequence data files for the 16S rRNA and 18S rRNA are available in the NCBI Sequence Read Archive (SRA) under Bioproject number PRJNA755150. All remaining data presented in this study are available on reasonable request from the corresponding author.

## Supplementary Materials

The following are available online at https://www.mdpi.com/article/10.3390/microorganisms9091891/s1, Figure S1: Regression analysis of the relative abundance of DMSP-degrading genes and *Sulfitobacter* abundance for the inner (red) and outer (yellow) reef sites, Table S1: Forward and reverse primer pairs for DMSP catabolizing genes and 16S normalisation gene, Table S2: Macronutrient concentrations for control and DMSP-enriched samples during experiment 2 (IR), Table S3: Macronutrient concentrations for control and DMSP-enriched samples during experiment 2 (OR), Table S4: SIMPER dissimilarity output for control and +DMSP treatment over time for inner reef (IR) and outer reef (OR) bacterioplankton (16S) and phytoplankton (18S) communities.

## References

[1] Simó R. (2004). From cells to globe: Approaching the dynamics of DMS(P) in the ocean at multiple scales. Can. J. Fish. Aquat. Sci..

[2] Keller M.M.D., Bellows W.W.K., Guillard R.R.R.L. (1989). Dimethyl Sulfide Production in Marine Phytoplankton. Biogenic Sulfur in the Environment.

[3] Matrai P.A., Keller M.D. (1994). Total organic sulfur and dimethylsulfoniopropionate in marine phytoplankton: Intracellular variations. Mar. Biol..

[4] Caruana A.M.N., Steinke M., Turner S.M., Malin G. (2012). Concentrations of dimethylsulphoniopropionate and activities of dimethylsulphide-producing enzymes in batch cultures of nine dinoflagellate species. Biogeochemistry.

[5] Keller M.D., Kiene R.P.R., Matrai P.A.P., Bellows W.K. (1999). Production of glycine betaine and dimethylsulfoniopropionate in marine phytoplankton. II. N-limited chemostat cultures. Mar. Biol..

[6] Simó R., Archer S.D., Pedrós-Alió C., Gilpin L., Stelfox-Widdicombe C.E. (2002). Coupled dynamics of dimethylsulfoniopropionate and dimethylsulfide cycling and the microbial food web in surface waters of the North Atlantic. Limnol. Oceanogr..

[7] Zubkov M., Fuchs B., Archer S., Kiene R.P., Amann R., Burkill P.H. (2001). Linking the composition of bacterioplankton to rapid turnover of dissolved dimethylsulphoniopropionate in an algal bloom in the North Sea. Environ. Microbiol..

[8] Karsten U., Kück K., Vogt C., Kirst G., Kiene R.P., Visscher P.T., Keller M.D., Kirst G.O., Visscher P.T. (1996). Dimethylsulfoniopropionate production in phototrophic organisms and its physiological functions as a cryoprotectant. Biological and Environmental Chemistry of DMSP and Related Sulfonium Compounds.

[9] Dickson D., Kirst G. (1986). The role of β-dimethylsulphoniopropionate, glycine betaine and homarine in the osmoacclimation of Platymonas subcordiformis. Planta.

[10] Sunda W., Kieber D.J., Kiene R.P., Huntsman S. (2002). An antioxidant function for DMSP and DMS in marine algae. Nature.

[11] Saha M., Rempt M., Gebser B., Grueneberg J., Pohnert G., Weinberger F. (2012). Dimethylsulphopropionate (DMSP) and proline from the surface of the brown alga Fucus vesiculosus inhibit bacterial attachment. Biofouling.

[12] Strom S., Wolfe G., Slajer A., Lambert S., Clough J. (2003). Chemical defense in the microplankton II: Inhibition of protist feeding by β-dimethylsulfoniopropionate (DMSP). Limnol. Oceanogr..

[13] Wolfe G.V., Steinke M., Kirst G.O. (1997). Grazing-activated chemical defence in a unicellular marine alga. Nature.

[14] Hill R., White B., Cottrell M., Dacey J. (1998). Virus-mediated total release of dimethylsulfoniopropionate from marine phytoplankton: A potential climate process. Aquat. Microb. Ecol..

[15] Matrai P.A., Keller M.D. (1993). Dimethylsulfide in a large-scale coccolithophore bloom in the Gulf of Maine. Cont. Shelf Res..

[16] Dacey J.W.H., Wakeham S.G. (1986). Oceanic Dimethylsulfide: Production During Zooplankton Grazing on Phytoplankton. Science.

[17] Malmstrom R.R., Kiene R.P., Kirchman D.L. (2004). Identification and enumeration of bacteria assimilating dimethylsulfoniopropionate (DMSP) in the North Atlantic and Gulf of Mexico. Limnol. Oceanogr..

[18] Ruiz-González C., Galí M., Sintes E., Herndl G.G.J., Gasol J.J.M., Simó R., Neilson A., Lewin R., Amblard C., Wright R. (2012). Sunlight Effects on the Osmotrophic Uptake of DMSP-Sulfur and Leucine by Polar Phytoplankton. PLoS ONE.

[19] Curson A.R.J., Todd J.D., Sullivan M.J., Johnston A.W.B. (2011). Catabolism of dimethylsulphoniopropionate: Microorganisms, enzymes and genes. Nat. Rev. Microbiol..

[20] Reisch C.R., Stoudemayer M.J., Varaljay V.A., Amster I.J., Moran M.A., Whitman W.B. (2011). Novel pathway for assimilation of dimethylsulphoniopropionate widespread in marine bacteria. Nature.

[21] Kiene R.P., Linn L.J., Bruton J.A. (2000). New and important roles for DMSP in marine microbial communities. J. Sea Res..

[22] Kiene R.P., Linn L.J., González J., Moran M.A., Bruton J.A. (1999). Dimethylsulfoniopropionate and methanethiol are important precursors of methionine and protein-sulfur in marine bacterioplankton. Appl. Environ. Microbiol..

[23] Kiene R.P., Linn L.J. (2000). The fate of dissolved dimethylsulfoniopropionate (DMSP) in seawater: Tracer studies using 35S-DMSP. Geochim. Cosmochim. Acta.

[24] Yoch D.C. (2002). Dimethylsulfoniopropionate: Its Sources, Role in the Marine Food Web, and Biological Degradation to Dimethylsulfide. Appl. Environ. Microbiol..

[25] Moran M.A., Reisch C.R., Kiene R.P., Whitman W.B. (2012). Genomic Insights into Bacterial DMSP Transformations. Ann. Rev. Mar. Sci..

[26] Simó R. (2001). Production of atmospheric sulfur by oceanic plankton: Biogeochemical, ecological and evolutionary links. Trends Ecol. Evol..

[27] Gao C., Fernandez V.I., Lee K.S., Fenizia S., Pohnert G., Seymour J.R., Raina J.-B., Stocker R. (2020). Single-cell bacterial transcription measurements reveal the importance of dimethylsulfoniopropionate (DMSP) hotspots in ocean sulfur cycling. Nat. Commun..

[28] Howard E.C., Henriksen J.R., Buchan A., Reisch C.R., Burgmann H., Welsh R., Ye W., Gonzalez J.M., Mace K., Joye S.B. (2006). Bacterial Taxa That Limit Sulfur Flux from the Ocean. Science.

[29] Howard E.C., Sun S., Biers E.J., Moran M.A. (2008). Abundant and diverse bacteria involved in DMSP degradation in marine surface waters. Environ. Microbiol..

[30] Todd J.D., Curson A.R.J., Dupont C.L., Nicholson P., Johnston A.W.B. (2009). The dddP gene, encoding a novel enzyme that converts dimethylsulfoniopropionate into dimethyl sulfide, is widespread in ocean metagenomes and marine bacteria and also occurs in some Ascomycete fungi. Environ. Microbiol..

[31] Choi D.H., Park K.-T., An S.M., Lee K., Cho J.-C., Lee J.-H., Kim D., Jeon D., Noh J.H. (2015). Pyrosequencing revealed SAR116 clade as dominant dddP-containing bacteria in oligotrophic NW Pacific Ocean. PLoS ONE.

[32] Sun J., Todd J.D., Thrash J.C., Qian Y., Qian M.C., Temperton B., Guo J., Fowler E.K., Aldrich J.T., Nicora C.D. (2016). The abundant marine bacterium Pelagibacter simultaneously catabolizes dimethylsulfoniopropionate to the gases dimethyl sulfide and methanethiol. Nat. Microbiol..

[33] Vila-Costa M., Simó R., Harada H., Gasol J.M., Slezak D., Kiene R.P. (2006). Dimethylsulfoniopropionate uptake by marine phytoplankton. Science.

[34] Petrou K., Nielsen D.A. (2018). Uptake of dimethylsulphoniopropionate (DMSP) by the diatom Thalassiosira weissflogii: A model to investigate the cellular function of DMSP. Biogeochemistry.

[35] Spielmeyer A., Gebser B., Pohnert G. (2011). Investigations of the uptake of dimethylsulfoniopropionate by phytoplankton. ChemBioChem.

[36] Ruiz-González C., Simó R., Vila-Costa M., Sommaruga R., Gasol J.M. (2012). Sunlight modulates the relative importance of heterotrophic bacteria and picophytoplankton in DMSP-sulphur uptake. ISME J..

[37] Theseira A.M., Nielsen D.A., Petrou K. (2020). Uptake of dimethylsulphoniopropionate (DMSP) reduces free reactive oxygen species (ROS) during late exponential growth in the diatom Thalassiosira weissflogii grown under three salinities. Mar. Biol..

[38] Alcolombri U., Ben-Dor S., Feldmesser E., Levin Y., Tawfik D.S., Vardi A. (2015). Marine sulfur cycle. Identification of the algal dimethyl sulfide-releasing enzyme: A missing link in the marine sulfur cycle. Science.

[39] Malin G. (2006). New pieces for the Marine Sulfur Cycle Jigsaw. Science.

[40] Wang S., Maltrud M.E., Burrows S.M., Elliott S.M., Cameron-Smith P. (2018). Impacts of Shifts in Phytoplankton Community on Clouds and Climate via the Sulfur Cycle. Glob. Biogeochem. Cycles.

[41] McCoy D.T., Burrows S.M., Wood R., Grosvenor D.P., Elliott S.M., Ma P.-L., Rasch P.J., Hartmann D.L. (2015). Natural aerosols explain seasonal and spatial patterns of Southern Ocean cloud albedo. Sci. Adv..

[42] Blondeau-Patissier D., Ernesto Brando V., Lønborg C., Leahy S.M., Dekker A.G. (2018). Phenology of trichodesmium spp. Blooms in the great barrier reef lagoon, Australia, from the ESA-MERIS 10-year mission. PLoS ONE.

[43] Revelante N., Williams W.T., Bunt J.S. (1982). Temporal and spatial distribution of diatoms, dinoflagellates and trichodesmium in waters of the Great Barrier Reef. J. Exp. Mar. Bio. Ecol..

[44] Westberry T.K., Siegel D.A. (2006). Spatial and temporal distribution of *Trichodesmium* blooms in the world’s oceans. Glob. Biogeochem. Cycles.

[45] Partensky F., Blanchot J., Vaulot D., Charpy L., Larkum A. (1999). Differential distribution and ecology of Prochlorococcus and Synechococcus in oceanic waters: A review. Marine Cyanobacteria.

[46] Broadbent A., Jones G. (2006). Seasonal and Diurnal Cycles of Dimethylsulfide, Dimethylsulfoniopropionate and Dimethylsulfoxide at One Tree Reef Lagoon. Environ. Chem..

[47] Broadbent A.D., Jones G.B., Jones R.J. (2002). DMSP in Corals and Benthic Algae from the Great Barrier Reef. Estuar. Coast. Shelf Sci..

[48] Jones G., Curran M., Broadbent A., King S., Fischer E. (2007). Factors affecting the cycling of dimethylsulfide and dimethylsulfoniopropionate in coral reef waters of the Great Barrier Reef. Environ. Chem..

[49] Jones G., Curran M., Broadbent A., Bellwood O., Choat H., Saxena N. (1994). Dimethylsulphide in the South Pacific. Recent Advances in Marine Science and Technology 1994.

[50] Del Valle D.A., Slezak D., Smith C.M., Rellinger A.N., Kieber D.J., Kiene R.P. (2011). Effect of acidification on preservation of DMSP in seawater and phytoplankton cultures: Evidence for rapid loss and cleavage of DMSP in samples containing *Phaeocystis* sp.. Mar. Chem..

[51] Harada H., Rouse M.-A., Sunda W., Kiene R.P. (2004). Latitudinal and vertical distributions of particle-associated dimethylsulfoniopropionate (DMSP) lyase activity in the western North Atlantic Ocean. Can. J. Fish. Aquat. Sci..

[52] Marie D., Partensky F., Jacquet S., Vaulot D. (1997). Enumeration and Cell Cycle Analysis of Natural Populations of Marine Picoplankton by Flow Cytometry Using the Nucleic Acid Stain SYBR Green I. Appl. Environ. Microbiol..

[53] Gasol J.M., Del Giorgio P.A., Giorgio P.A. (2000). del Using flow cytometry for counting natural planktonic bacteria and understanding the structure of planktonic bacterial communities. Sci. Mar..

[54] Seymour J.R., Seuront L., Mitchell J.G. (2007). Microscale gradients of planktonic microbial communities above the sediment surface in a mangrove estuary. Estuar. Coast. Shelf Sci..

[55] Cowley R., Critchley G., Eriksen R., Latham V., Plaschke R., Rayner M., Terhell D. (1999). CSIRO Research Publications Repository—Hydrochemistry Operations Manual.

[56] Callahan B.J., McMurdie P.J., Rosen M.J., Han A.W., Johnson A.J., Holmes S.P. (2016). DADA2: High-resolution sample inference from Illumina amplicon data. Nat. Methods.

[57] Martin M. (2011). Cutadapt removes adapter sequences from high-throughput sequencing reads. EMBnet J..

[58] Levine N.M., Varaljay V.A., Toole D.A., Dacey J.W.H., Doney S.C., Moran M.A. (2012). Environmental, biochemical and genetic drivers of DMSP degradation and DMS production in the Sargasso Sea. Environ. Microbiol..

[59] Varaljay V.A., Howard E.C., Sun S., Moran M.A. (2010). Deep sequencing of a dimethylsulfoniopropionate-degrading gene (dmdA) by using PCR primer pairs designed on the basis of marine metagenomic data. Appl. Environ. Microbiol..

[60] Suzuki M.T., Taylor L.T., DeLong E.F. (2000). Quantitative analysis of small-subunit rRNA genes in mixed microbial populations via 5′-nuclease assays. Appl. Environ. Microbiol..

[61] Clarke K.R., Gorley R.N. PRIMER V6 Software.

[62] R Core Team (2021). R: A Language and Environment for Statistical Computing.

[63] Wickham H., Averick M., Bryan J., Chang W., McGowan L., François R., Grolemund G., Hayes A., Henry L., Hester J. (2019). Welcome to the Tidyverse. J. Open Source Softw..

[64] Oksanen J., Blanchet F., Guillaume Friendly M., Kindt R., Legendre P., McGlinn D., Minchin R.P., O’Hara R.B., Simpson G.L., Solymos P. Vegan: Community Ecology Package 2020. https://cran.r-project.org/web/packages/vegan/index.html.

[65] Kassambara A. Ggpubr: “Ggplot2” Based Publication Ready Plots 2020. https://cran.r-project.org/web/packages/ggplot2/index.html.

[66] Kiene R.P., Linn L.J. (2000). Distribution and turnover of dissolved DMSP and its relationship with bacterial production and dimethylsulfide in the Gulf of Mexico. Limnol. Oceanogr..

[67] Andrews J.C. (1983). Water masses, nutrient levels and seasonal drift on the outer central queensland shelf (great barrier reef). Mar. Freshw. Res..

[68] Crosbie N.D., Furnas M.J. (2001). Abundance, distribution and flow-cytometric characterization of picophytoprokaryote populations in central (17°S) and southern (20°S) shelf waters of the Great Barrier Reef. J. Plankton Res..

[69] Muslim I., Jones G. (2003). The seasonal variation of dissolved nutrients, chlorophyll a and suspended sediments at Nelly Bay, Magnetic Island. Estuar. Coast. Shelf Sci..

[70] Brimblecombe P., Shooter D. (1986). Photo-oxidation of dimethylsulphide in aqueous solution. Mar. Chem..

[71] Visscher P., Diaz M., Taylor B. (1992). Enumeration of bacteria which cleave or demethylate dimethylsulfoniopropionate in the Caribbean Sea. Mar. Ecol. Prog. Ser..

[72] Zeyer J., Eicher P., Wakeham S.G., Schwarzenbach R.P. (1987). Oxidation of Dimethyl Sulfide to Dimethyl Sulfoxide by Phototrophic Purple Bacteria. Appl. Environ. Microbiol..

[73] Malmstrom R.R., Kiene R.P., Vila M., Kirchman D.L. (2005). Dimethylsulfoniopropionate (DMSP) assimilation by Synechococcus in the Gulf of Mexico and northwest Atlantic Ocean. Limnol. Oceanogr..

[74] Campbell L., Nolla H.A., Vaulot D. (1994). The importance of Prochlorococcus to community structure in the central North Pacific Ocean. Limnol. Oceanogr..

[75] Partensky F., Hess W.R., Vaulot D. (1999). Prochlorococcus, a marine photosynthetic prokaryote of global significance. Microbiol. Mol. Biol. Rev..

[76] Vila-Costa M., del Valle D.A., González J.M., Slezak D., Kiene R.P., Sánchez O., Simó R. (2006). Phylogenetic identification and metabolism of marine dimethylsulfide-consuming bacteria. Environ. Microbiol..

[77] Lei L., Alcolombri U., Tawfik D.S. (2017). DddY is a bacterial dimethylsulfoniopropionate lyase representing a new cupin enzyme superfamily with unknown primary function. bioRxiv.

[78] Varaljay V.A., Gifford S.M., Wilson S.T., Sharma S., Karl D.M., Moran M.A. (2012). Bacterial dimethylsulfoniopropionate degradation genes in the oligotrophic north pacific subtropical gyre. Appl. Environ. Microbiol..

[79] Malmstrom R.R., Kiene R.P., Cottrell M.T., Kirchman D.L. (2004). Contribution of SAR11 Bacteria to Dissolved Dimethylsulfoniopropionate and Amino Acid Uptake in the North Atlantic Ocean. Appl. Environ. Microbiol..

[80] Cui Y., Suzuki S., Omori Y., Wong S.-K., Ijichi M., Kaneko R., Kameyama S., Tanimoto H., Hamasaki K. (2015). Abundance and distribution of dimethylsulfoniopropionate degradation genes and the corresponding bacterial community structure at dimethyl sulfide hot spots in the tropical and subtropical pacific ocean. Appl. Environ. Microbiol..

[81] Liu J., Liu J., Zhang S.-H., Liang J., Lin H., Song D., Yang G.-P., Todd J.D., Zhang X.-H. (2018). Novel Insights Into Bacterial Dimethylsulfoniopropionate Catabolism in the East China Sea. Front. Microbiol..

[82] Motard-Côté J., Kiene R. (2015). Osmoprotective role of dimethylsulfoniopropionate (DMSP) for estuarine bacterioplankton. Aquat. Microb. Ecol..

[83] Simó R., Pedrós-Alió C., Malin G., Grimalt J. (2000). Biological turnover of DMS, DMSP and DMSO in contrasting open-sea waters. Mar. Ecol. Prog. Ser..

[84] Simó R., Pedrós-Alió C. (1999). Short-term variability in the open ocean cycle of dimethylsulfide. Glob. Biogeochem. Cycles.

[85] Wolfe G., Steinke M. (1996). Grazing-activated production of dimethyl sulfide (DMS) by two clones of Emiliania huxleyi. Limnol. Oceanogr..

[86] Van Boekel J., Stefels W. (1993). Production of DMS from dissolved DMSP in axenic cultures of the marine phytoplankton species Phaeocystis sp.. Mar. Ecol. Prog. Ser..

[87] Yost D.M., Mitchelmore C.L. (2009). Dimethylsulfoniopropionate (DMSP) lyase activity in different strains of the symbiotic alga symbiodinium microadriaticum. Mar. Ecol. Prog. Ser..

[88] Yost D.M., Mitchelmore C.L. (2012). Substrate kinetics of DMSP-lyases in various cultured symbiodinium strains. Bull. Mar. Sci..

[89] Nowinski B., Motard-Côté J., Landa M., Preston C.M., Scholin C.A., Birch J.M., Kiene R.P., Moran M.A. (2019). Microdiversity and temporal dynamics of marine bacterial dimethylsulfoniopropionate genes. Environ. Microbiol..

