# Supplementary material for: Uptake of Dimethylsulfoniopropionate (DMSP) by Natural Microbial Communities of the Great Barrier Reef (GBR), Australia
Source: Microorganisms. 2021 Sep 6;9(9):1891. doi: 10.3390/microorganisms9091891 (PMC8471478; doi:10.3390/microorganisms9091891)
Supplement: Supplementary file 1 [file microorganisms-09-01891-s001.zip › microorganisms-1276251-supplementary.pdf]

SUPPLEMENTARY FILE

Uptake of dimethylsulfoniopropionate (DMSP) by natural microbial communities of the Great Barrier Reef (GBR), Australia

Fernandez E[1,2], Ostrowski M[2], Siboni N[2], Seymour JR[2], Petrou K[1]*

[1] School of Life Sciences, University of Technology Sydney, Sydney, NSW, Australia

[2] Climate Change Cluster, University of Technology Sydney, Sydney, NSW, Australia

Supplementary Table S1. Forward and reverse primer pairs for three DMSP catabolising genes and 16S normalisation gene.

Primer name	Primer sequence	Annealing temperature	Reference
**DmdA/A1**	A/1-spFP, 5′- ATGGTGATTTGCTTCAGTTTCT -3′ and A/1-spRP 5′-CCCTGCTTTGACCAACC -3′	54°C	Varaljay et al., 2010
**DddP**	dddP_874F 5′- AAYGAAATWGTTGCCTTTGA -3′ and dddP_971R 5′- GCATDGCRTAAATCATATC -3′	41°C	Levine et al., 2012
**DmdA/Dall**	D/all-spFP 5′ -TATTGGTATAGCTATGAT- 3′ and D/all-spRP 5′- TAAATAAAAGGTAAATCGC-3′	42°C	Varaljay et al., 2010
**16S**	BACT- 1369F 5′- CGGTGAATACGTTCYCGG -3′ and PROK- 1492R 5′- GGWTACCTTGTTACGGACTT -3′	53°C	Suzuki et al., 2000*

* 16S assay was used without the TaqMan probe and was a 2-step assay. All other assays were 3-step assays

Supplementary Table S2. Macronutrient concentrations for control and DMSP-enriched samples during experiment 2. Inner reef macronutrient concentrations over 120h. Macronutrient concentrations are in μM. BDL = below detection limit

Treatment	0h	24h	72h	120h
NOx				
Control	BDL	0.04	0.13	BDL
DMSP	BDL	0.07	0.09	0.03
Silicate				
Control	0.5	0.8	1.80	0.90
DMSP	0.5	1.6	2.00	0.97
Ammonium				
Control	BDL	0.00	0.04	BDL
DMSP	BDL	0.00	BDL	BDL
Phosphate				
Control	0.05	0.04	0.04	BDL
DMSP	0.05	0.04	0.07	BDL

Supplementary Table S3. Macronutrient concentrations for control and DMSP-enriched samples during experiment 2. Outer reef macronutrient concentrations over 120h. Macronutrient concentrations are in µM. BDL = below detection limit

Treatment	0h	24h	72h	120h
NOx				
Control	BDL	No data	0.18	0.10
DMSP	BDL	No data	0.16	0.06
Silicate				
Control	0.882	No data	1.00	1.60
DMSP	0.882	No data	1.10	1.37
Ammonium				
Control	BDL	No data	BDL	BDL
DMSP	BDL	No data	0.17	0.073
Phosphate				
Control	0.066	No data	0.08	0.070
DMSP	0.066	No data	0.07	0.067

Supplementary Table S4: SIMPER dissimilarity output for control and +DMSP treatments over time for the inner reef (IR) and outer reef (OR) bacterioplankton (16S) and phytoplankton (18S) communities. Only the highest contributing taxa (order level) contributing to >50% or the total dissimilarity are presented.

**IR Bacterioplankton total dissimilarity**	**19.85%**			
Taxa	*Control Av Abundance*	*+DMSP Av Abundance*	*Contribution (%)*	*Cumulative contribution (%)*
Rhodobacterales	48.04	29.31	21.75	21.75
Synechococcales	46.87	54.22	10.22	31.97
Actinomarinales	16.80	26.07	9.35	41.32
SAR11 clade	38.80	44.42	7.08	48.40
Rhodospirillales	18.43	22.86	6.19	54.59
**OR Bacterioplankton total dissimilarity**		**19.40%**		
Rhodobacterales	41.82	63.95	18.67	18.67
Synechococcales	55.00	38.36	14.10	32.77
Actinomarinales	12.87	2.76	11.61	44.38
Sphingomonadales	5.28	10.68	9.23	53.61
**IR Phytoplankton total dissimilarity**		**22.83%**		
Diatoms [centrales]	75.66	84.12	14.27	14.27
Dino-Group [I, II, III, V]	191.8	170.9	11.60	25.87
Gymnodiniales	133.3	129.0	9.70	35.57
Suessiales	28.85	17.53	8.79	44.36
Diatoms [pennales]	18.48	31.22	7.06	51.42
**OR Phytoplankton total dissimilarity**		**26.46%**		
Dino-Group [I, II, III, V]	222.2	172.9	18.47	18.47
Diatoms [centrales]	40.99	0.00	12.40	30.87
MAST-group	80.32	63.91	8.20	39.07
Gymnodiniales	118.9	125.8	7.26	46.33
Stramenopiles	4.67	29.24	7.16	53.49

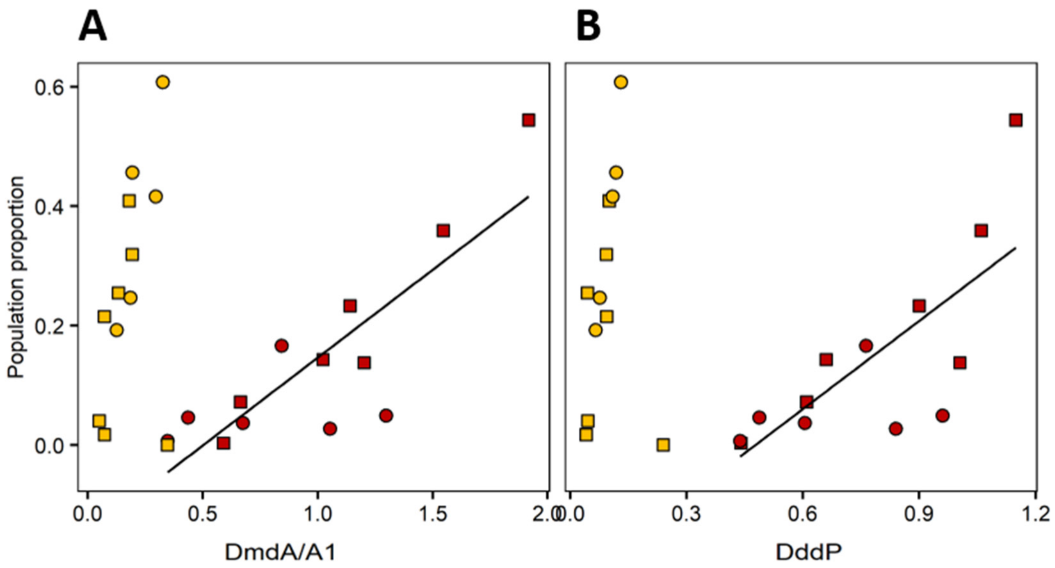

Supplementary Figure S1. Regression analysis of the relative abundance of DMSP-degrading genes and *Sulfitobacter* abundance for inner (red) and outer (yellow) reef sites. The relative abundance of (A) *Sulfitobacter* and DmdA/A1, (B) *Sulfitobacter* and DddP. Squares, controls; circles, +DMSP. Lines show significant linear regressions (A: $_{adj}R^2=0.67$, $P<0.0003$; B: $_{adj}R^2=0.52$, $P<0.0032$).
